# Dapagliflozin and atrial fibrillation: elevated dosing to achieve class I antiarrhythmic effects?

**DOI:** 10.1007/s00395-024-01047-z

**Published:** 2024-04-03

**Authors:** Torsten Christ, Edzard Schwedhelm, Thomas Eschenhagen

**Affiliations:** 1 Institute of Experimental Pharmacology and Toxicology, University Medical Center Hamburg-Eppendorf, Martinistraße 52, 20246 Hamburg, Germany; 2 DZHK (German Centre for Cardiovascular Research), Partner Site Hamburg/Kiel/Lübeck, Hamburg, Germany; 3 Institute of Clinical Pharmacology and Toxicology, University Medical Center Hamburg-Eppendorf, Hamburg, Germany

Inhibitors of sodium–glucose co-transporter-2 (SGLT2i) robustly decrease morbidity and mortality in patients with heart failure and chronic kidney disease, both in diabetic and non-diabetic patients. Several mechanisms have been proposed to explain the unexpected cardio- and kidney-protective effect, including inhibition of the cardiac sodium-hydrogen exchanger [16], increase in renal production of erythropoietin, their ketogenic action and a simple diuretic effect [13]. SGLT2i have also been shown to inhibit the pathological late sodium current, a current initially identified in LQT3 and then as a maladaptive mechanism in chronic heart failure [14]. Concentration-dependent inhibition of late sodium current by several SGLT2i was observed at 0.5–1 µmol/L, representing the higher range of clinically relevant concentrations, at which no inhibition of peak sodium current was noted. Other data indicate that the effect of SGLT2i on late sodium influx might occur indirectly via inhibition of CaMKII [10]. Moreover, the action seems not to be a universal mechanism, since SGLT2i were devoid of any effect on late sodium currents in a study on induced pluripotent stem cell derived-cardiomyocytes with a hypertrophic cardiomyopathy background [16].

SGLT2i may also be cardioprotective by interfering with the complex mechanisms of myocardial ischemia/reperfusion injury [7]. In fact, 1 week treatment with empagliflozin or dapagliflozin, but not with ertugliflozin (another SGLT2i), reduced myocardial ischemia/reperfusion injury in healthy non-diabetic mice, indicating “off-target” effects of distinct SGLT2i’s [11]. Empagliflozin also ameliorated anthracycline-associated cardiotoxicity [15] leading to the multicenter phase 3 study EMPACT (NCT05271162). Finally, a recent unbiased proteomics and metabolomics study showed that SGLT2i mainly affect the gut microbiome and the kidney, where they induce less production and greater excretion of amino acid metabolites (“uremic toxins”) with cardiodepressant effects [2].

Several randomized controlled trials demonstrate that SGLT2 inhibition is associated with significantly reduced incidence of atrial arrhythmias (AF) and sudden cardiac death in patients with type 2 diabetes mellitus or heart failure [4]. This clinical benefit has initiated a wide research on the question how SGLT2 inhibitors may mediate their antiarrhythmic effects. Several putative pharmacodynamics were identified, many of them involved in the regulation of intracellular calcium [5]. In this context, we read with interest the paper by Paasche et al.[12], which proposes SGLT2i as a new treatment option in AF. Unexpected for us was the pharmacodynamics the authors suspected to be operative: block of peak cardiac sodium currents. The authors measured blocking effects of dapagliflozin on cardiac sodium channels in Chinese hamster ovary (CHO) cells expressing human SCNA5 (encoding the cardiac sodium channel) and in ventricular and atrial hiPSC-cardiomyocytes (CM). While exact IC_50_ values for dapagliflozin block of SCNA5 in CHO cells were not given, Fig. 3G indicates an IC_50_ value of at least 30 µmol/L. Concentration–response curves were given for dapagliflozin block of sodium currents in hiPSC-CM. However, the curves are based on three concentrations only, despite the fact that the authors used an automated patch clamp system that should allow high throughput. Furthermore, the block of sodium currents in hiPSC-CM by dapagliflozin is by far not complete, complicating interpretation of IC_50_ values. In a second part, the authors measured AP in pig atrial, human atrial and hiPSC-derived atrial CM. Cells were injected with a short depolarizing pulse of fixed amplitude. The inducibility of an AP was expressed as the “percentage of pulses evoking an AP, out of 10 current pulses”. Dapagliflozin decreased the numbers of AP elicited. Such an attempt is uncommon and not a validated method to determine effects of sodium channel blockers. The interpretation is further complicated by the fact that, for reasons not given, holdings currents were applied. Anyhow, at least 10 µmol/L was needed in adult human atrial or 30 µmol/L in hiPSC-aCM to achieve threshold effects on excitability.

Dapagliflozin underwent rigorous safety testing previously. Even at a supra-therapeutic dose (150 mg instead of the standard dose of 10 mg once daily), QRS duration was not prolonged in healthy volunteers [3]. In this safety study, C_max_ was 4.8 µmol/L, which, at a plasma protein-binding rate of 90%, translates into a free plasma concentration of 0.48 µmol/L. In the same study, the highest FDA-approved dose of 20 mg/day dapagliflozin, resulted in a total plasma concentration of 0.61 µmol/L, corresponding to a free plasma concentration of approx. 0.06 µmol/L, i.e. 500-fold lower than the IC_50_ of dapagliflozin to block sodium channels of 30 µmol/L reported in Paasche et al.[12]. Even though free plasma concentrations of dapagliflozin are slightly higher in patients treated with multiple doses, i.e. in the steady state, this precludes relevant sodium channel block at therapeutically relevant plasma concentrations of dapagliflozin. The same conclusion can be drawn from the first study relating effects of SGLT2i with sodium channel block [14], where empagliflozin and dapagliflozin (10 µmol/L) were devoid of effects on peak sodium current (while they had an effect on the pathological late sodium current). Thus, we would not expect that the potential beneficial effects by dapagliflozin in AF are mediated via sodium channel block, the main conclusion of the study by Paasche et al. [12].

The reduction of excitability by sodium channel block is a classic antiarrhythmic concept in cardiology, proven at great detail both experimentally and clinically. Thus, sodium channel block can undoubtedly stop AF [1], but it is also well established that sodium channel block can slow electrical conductance in the ventricle and thereby increase the propensity for potentially life-threatening arrhythmias in hearts with structural pathologies [8]. Thus, inhibition of the peak sodium current by SGLT2i would represent a substantial risk, particularly in patients with heart failure, an established indication for the use of this class of drugs. As a result, there is high awareness for this problem. Concerted activities of leading pharmaceutical companies have led to sophisticated strategies how to refine screening for potential unwanted sodium channel effects of drugs [6]. According to these algorithms, SGLT2i must be considered safe drugs, because the ratio of the IC_50_ at sodium channels (peak current) and free therapeutic plasma concentrations is > 100. This safety margin is well compatible with the absence of pro-arrhythmic signals in clinical studies in patients with heart failure [9].

Thus, we doubt that inhibitory effects of SGLT2 inhibitors on the sodium channel at concentrations several fold higher than those reached at therapeutic doses may open new treatment modalities in AF. Nor do we believe that the new data by Paasche et al. raise a relevant safety concern.
